# Lipohypertrophy in children and adolescents with type 1 diabetes and the associated factors

**DOI:** 10.1186/1756-0500-4-290

**Published:** 2011-08-12

**Authors:** Magdy A Omar, Ahmed A El-Kafoury, Ramy I El-Araby

**Affiliations:** 1 El-Chatby University Children's Hospital, Department Of Paediatrics, Alexandria University, Egypt

## Abstract

**Background:**

Despite the important implications of lipohypertrophy for diabetes control, there is a dearth of information and research about the subject in children. The aim of this study was to study the prevalence of lipohypertrophy in children with type 1 diabetes, and to evaluate the associated factors.

**Findings:**

119 children coming for regular follow up in the diabetes clinic were examined for the presence of lipohypertrophy by inspection and palpation. The last 4 readings of glycated hemoglobin (HbA1c) levels and other factors that may affect lipohypertrophy were documented. RESULTS: The patient's age ranged from 2 months to 21 years with a median of 10 years (inter-quartile range = 6). Lipohypertrophy occurred in 54.9% of patients, more commonly in males (62.7%) vs. females (48.4%) (P = 0.074). Grade 1 lipohypertrophy occurred in 42.5% and grade 2 in 12.4%. Lipohypertrophy was related significantly to the dose of insulin units per kg of body weight (Odds ratio [OR] = 16.4; 95% CI, 2.2 - 124.6; P = 0.007), the duration of diabetes, [OR] = 1.16; 95% CI, 1.05 - 1.32; P = 0.004)), and the body mass index (BMI) [OR] = 1.68; 95% CI, 1.25 - 2.15; P = 0.006). The mean HbA1c levels of patients with grade 1 and grade 2 lipohypertrophy did not differ from diabetics without lipohypertrophy (F = 0.178, P = 0.837)

**Conclusions:**

The presence of lipohypertrophy was significantly associated with the duration of diabetes and the body mass index. Children with lipohypertrophy needed a significantly higher dose of insulin units/kg of body weight to achieve fair control compared to children without lipohypertrophy. Further studies are needed to ascertain the clinical meaning of these findings.

## Findings

Lipohypertrophy is a frequent problem in young patients with type 1 diabetes, occurring in up to 50% of patients, it may be associated with poor glycaemic control, [1] Although a cause for these lesions is not known, the predisposing conditions may be trauma to the skin and subcutaneous tissue repeated over time in the presence of insulin [2]. Histolgically the hypertrophic adipocytes are twice as large as those from normal subcutaneous areas and contained numerous small lipid droplets. Electron microscopic analysis also revealed a minor population of small adipocytes, suggesting active differentiation or proliferation [3]. The injection of insulin into a site of lipohypertrophy may lead to erratic absorption of the insulin, with the potential for poor glycaemic control and unpredictable hypoglycaemia [4]. Despite the important implications for diabetes control in insulin-injecting patients, there is a dearth of information in children about the subject. The aim of this study was to evaluate the prevalence of lipohypertrophy in children with type 1diabetes, to study parameters associated with its development and to assess its effect on diabetes control.

## Methods

Patients were evaluated and examined for the presence of lipohypertrophy at routine follow-up clinic visits at the Diabetes Clinic for children at El-Chatby University Children's Hospital, Alexandria. (A free teaching hospital). Observation and palpation techniques were used in assessing lipohypertrophy in these diabetics. Lipohypertrophy was graded as following: grade 0 = no changes; grade 1 = visible hypertrophy of fat tissue but palpably normal consistency; grade 2 = massive thickening of fat tissue with firm consistency; and grade 3 = lipoatrophy [2]. Their age, weight, height, adjusted body mass index, duration of diabetes, HbA1c levels (average of the last 4 readings), needle length, use of syringes, pen, number of daily injections, washing hands before injection, insulin regimen, individuals giving the injection and education of their mothers were documented. Patients and mothers were asked how they rotate the injection sites. Patients received human insulin from diabetes onset. Most patients received a mixture of NPH and regular insulin 30/70, only 7 received glargine insulin. Patients were taught to rotate their injection sites every day according to a scheme (left, right arm/left, right thigh and/or left, right abdominal area). Data was analyzed using the Statistical Package for the Social Sciences (SPSS 14.0). Differences between the groups were calculated by the chi-square test for categorical variables, ANOVA test for two or more continuous variables, and logistic regression to obtain odds ratios for variables with significant P-values. The study protocol was approved by the institutional review board of the college of medicine in Alexandria University (Egypt), and a written parental consent and child assent were obtained before the study.

## Results

### 1. Prevalence of lipohypertrophy

The injection sites were examined in 119 children during their regular visits to the diabetes clinic (64 female and 55 male). Their ages ranged from 8 months to 21 years with a median of 10 years (inter-quartile range = 6). Prevalence of lipohypertrophy in patients injecting in the arms was: no change in 45.1%, grade 1 lipohypertrophy in 42.5% and grade 2 lipohypertrophy in 12.4% (table 1). The thighs were used for injecting in 63% of children and showed no change in 76%, grade 1 lipohypertrophy in 16% and grade 2 lipohypertrophy in 8% (Table 1). The thighs were used less frequently as an injection site, lipohypertrophy was not seen in the abdomen, as 83% of the patients were afraid to inject in the abdomen. lipohypertrophy was not affected significantly by rotation of the injection site (P = 0.74).

**Table 1:** Prevalence of lipohypertrophy at different injection sites

	Grade 0 Lipohypertrophy (n = 51)		Grade1 Lipohypertrophy (n = 48)		Grade2 Lipohypertrophy (n = 14)		Total (n = 113)	
	No	%	No	%	No	%	No	
**The arms**	51	45.1	48	42.5	14	12.4	113	
**The thighs**	57	76	12	16	6	8	75	
**The abdomen**	14	100	0	0	0	0	14	

### 2. Factors associated with lipohypertrophy

About 69% of patients were using micro fine needle (8 mm length), Lipohypertrophy occured in 48.7% of them. The pen was used in 13.3% of cases and lipohypertrophy occurred in 73.45%, this was significant by chi-square test (P = 0.043), but on doing logistic regression, the Injection method was not associated significantly with lipohypertrophy.

Percentile adjusted BMI was calculated and was found that 76.1% of children were normal, 15.0% overweight, 3.5% obese and 5.3% were under weight. This finding was significantly related to the occurrence of lipohypertrophy [OR] = 1.68; 95% CI, 1.25 - 2.15; P = 0.006. (Table 2)

**Table 2:** Logistic regression for factors with a significant P-value

	B	**S.E**.	df	P	OR	**95% C.I**.
**D.M Duration**	0.15	0.06	1	0.004	1.16	1.05 - 1.32
**BMI**	0.52	0.05	1	0.006	1.68	1.25 - 2.15
**Insulin units/kg**	2.8	1.0	1	0.007	16.4	2.2 - 124.6
**Constant**	-0.68	0.34	1	0.048	0.509	

P value for the model = 0.004

All Patients with grade, 1 and 2 lipohypertrophy had HbA1c levels similar to patients without lipohypertrophy as the mean was 8.4 ± 2.1, 8.8 ± 1.9, 8.5 ± 2.3 respectively. This was not statistically significant (P = 0.837), but lipohypertrophy was related significantly to the dose of insulin units per kg of body weight as the mean was 0.75 ± 0.21 U/kg in patients without lipohypertrophy versus 0.99 ± 0.52 U/kg in patients with grade 2 lipohypertrophy [OR] = 16.4; 95% CI, 2.2 - 124.6; P = 0.007. Lipohypertrophy was related also to the duration of diabetes, as the mean duration was 4.13 ± 3.67 years in patients with grade 0 and 7.07 ± 2.841 years in grade 2 lipohypertrophy [OR] = 1.16; 95% CI, 1.05 - 1.32; P = 0.004. In one patient lipohypertrophy developed 6 months after the onset of diabetes.

Washing hands before injections, type of insulin, number of injections per day, the frequency of changing the needle, the individual giving the injection, history of atopy, and the degree of mother's education, all did not affect the occurrence of lipohypertrophy significantly

## Discussion

Lipohypertrophy as a local complication of insulin therapy is well recognized, it is the most common cutaneous complication of insulin therapy, despite improvements in insulin purity and the introduction of recombinant human insulin its prevalence has remained high. The importance of this complication is not only cosmetic [5], but it also may impact on insulin absorption, and therefore its effect on glycaemic control is not well known.

In this study the prevalence of lipohypertrophy was similar to other studies [1,2], it occurred more in the arms, as the thighs were used less frequently as an injection site, children stated pain and bleeding after injection were the main reasons of not using the thighs, many children could not express the reasons, and this was mainly due to their young age. Lipohypertrophy in our patients was not significantly affected by rotation of the injection sites as many children without lipohypertrophy were not rotating injections sites despite repeated advice. In one patient lipohypertrophy developed 6 months after the onset of diabetes and this may denote that lipohypertrophy is not only caused by repeated trauma to the skin but other unknown factors exist.

It was observed that patients with lipohypertrophy needed a significantly higher dose of insulin units per kg of their body weight compared to those without lipohypertrophy, this could be due to defective absorption of insulin by the abnormal injection sites [6] or the high titre of insulin antibodies found in children with type 1 diabetes and lipohypertrophy [7]. It was concluded by Overland J, et al, [8] that "the pharmacokinetic and pharmacodynamic effect of injecting into lipohypertrophic tissue is small in comparison to the usual clinical variation observed with insulin injections as there was no difference in continuous glucose monitoring (CGMS) profiles between areas with lipohypertrophy and areas without lipohypertrophy. Therefore, the exact effect of lipohypertrophy is still not clear. Lipohypertrophy was significantly less common in obese and overweight versus normal and underweight patients, similar findings were observed by Conwell LS et al [9] but with continuous subcutaneous insulin infusion, this may be explained as in underweight and normal children the areas of lipohypertrophy are more easily seen and felt than in obese patients.

## Conclusion

The presence of lipohypertrophy was significantly associated with the duration of diabetes and the body mass index. Children with lipohypertrophy needed a significantly higher dose of insulin units/kg of body weight to achieve fair control compared to children without lipohypertrophy. Further studies are needed to ascertain the clinical meaning of these findings.

## List of abbreviations

HbA1c: Glycated haemoglobin; BMI: Body mass index.

## Competing interests

The authors declare that they have no competing interests.

## Authors' contributions

MAO revised the manuscript.AAK conceived, designed the study and wrote the manuscript and RIA helped in data collection. All authors read and approved the final manuscript.
